# Supplementary figures and images for: Deep learning with satellite images enables high-resolution income estimation: A case study of Buenos Aires
Source: PLoS One. 2026 Jan 16;21(1):e0338110. doi: 10.1371/journal.pone.0338110 (PMC12810835; doi:10.1371/journal.pone.0338110)

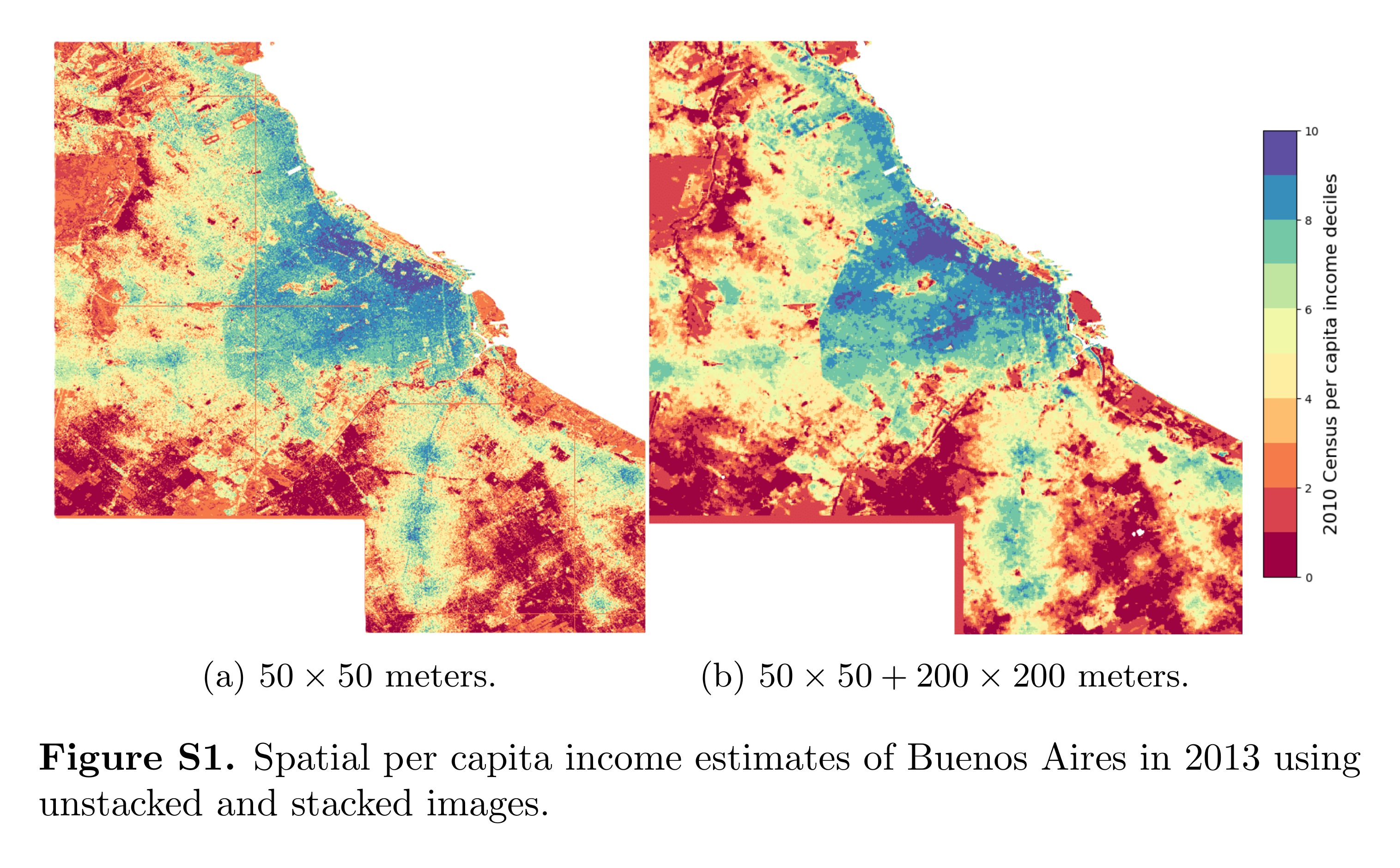

Supplement: S1 Fig — Spatial per capita income estimates of Buenos Aires in 2013 using unstacked and stacked (50x50m+200x200m) images. (TIF) [file pone.0338110.s002.tif]
