# Supplementary material for: Deep learning with satellite images enables high-resolution income estimation: A case study of Buenos Aires
Source: PLoS One. 2026 Jan 16;21(1):e0338110. doi: 10.1371/journal.pone.0338110 (PMC12810835; doi:10.1371/journal.pone.0338110)
Supplement: Attachment — Submitted filename: Response to Reviewers.pdf [file pone.0338110.s004.pdf]

Editorial Comments

> Thank you for submitting your manuscript to PLOS ONE. After careful consideration, we feel that it has merit but does not fully meet PLOS ONE's publication criteria as it currently stands. Therefore, we invite you to submit a revised version of the manuscript that addresses the points raised during the review process.

Reply:

Thank you for providing us with the opportunity to revise our manuscript, "Deep learning with satellite images enables high-resolution income estimation: a case study of Buenos Aires" (PONE-D-24-42689), and for the detailed feedback on journal requirements. We have carefully addressed each point as outlined below.

The main changes in the paper, compared to our previous version, are the following:

1. **Restructured discussion**. Former Section 7 was split into:
   - Section 7 – "Conceptualizing the Model's Measurement of Income," clarifying that the model targets *permanent* rather than short-term income;
   - Section 8 – "Generalizing the Model to Other Cities," with new content on fine-tuning, transferability, explainability, and ethics.
2. **New inequality metrics**. Theil and Atkinson (= 1, 2) indices were added to Table 4; Section 6.3 now contains an expanded primer on all inequality / poverty indicators (90/10, 50/10, Gini, FGT family, etc.).
3. **Additional robustness material**. A new footnote, Figure S1, and Table S1 demonstrate how stacking 50 × 50 m and 200 × 200 m tiles reduces spatial noise; Moran's I statistics at four distance bands are reported.
4. **Limitations emphasised**. New text highlights sensitivity to low-density areas, external imaging conditions, and the need for a two-stage model (household-presence → income) in sparsely populated zones.

Furthermore, some additional minor modifications where included:

5. The abstract now states the forecast years (2013, 2018, 2022).
6. The bibliography includes several 2024 studies on transformer-based remote-sensing models.
7. We have corrected a few spelling mistakes and writing inconsistencies.

8. We have added a footnote (page 2) about the relevance of the data we've employed, even if it is proprietary.
9. The MSE for the first two rows of Table 3 was corrected, as they were abnormally high due to a transcription error. Now those results are more in line with the other models.
10. We have explicitly incorporated the replication package (with the corresponding limitations) into the Supporting Information.
11. Figure 1 was updated to correct a small mistake in the order of sections 4.1 and 4.2; and Figure 6, the legend has been updated so the colors for the census tracts and the satellite images now match their intended assignments.

1. Please ensure that your manuscript meets PLOS ONE's style requirements, including those for file naming.

Reply:

We have reviewed our manuscript and the PLOS ONE style templates and believe that the revised manuscript fully adheres to the style requirements.

2. Please note that PLOS ONE has specific guidelines on code sharing for submissions in which author-generated code underpins the findings in the manuscript. In these cases, we expect all author-generated code to be made available without restrictions upon publication of the work. Please review our guidelines and ensure that your code is shared in a way that follows best practice and facilitates reproducibility and reuse.

Reply:

We understand and agree with PLOS ONE's guidelines on code sharing. All author-generated code that underpins the findings in this manuscript, including scripts for the Small Area Estimation (SAE) process, image preprocessing (such as stacking and pansharpening logic), CNN model training, and the generation of final income maps, are already available without restrictions on GitHub. We have explicitly included this repository as supporting information (S1 Replication Scripts).

3. We noted in your submission details that a portion of your manuscript may have been presented or published elsewhere. "As mentioned in the cover letter, an earlier version of this manuscript has been published in L CLEI 2024 conferences. Some figures of the methodology are fairly similar, as some of the decisions from such section remain the same. Nevertheless, all the results have been updated as there where many changes in the methodology, and the results are much more presice due to these updates. Furhtermore, many subsections of results have been added as the models have been tested much further than the earlier discussion manuscript." Please clarify whether this [conference proceeding or publication] was peer-reviewed and formally published. If this work was previously peer-reviewed and published, in the cover letter please provide the reason that this work does not constitute dual publication and should be included in the current manuscript.

Reply:

Thank you for the opportunity to clarify the previous presentation of portions of this work.

The manuscript presented at the "L CLEI 2024 conferences" underwent peer review and was formally published as conference proceedings. However, it represents an earlier, preliminary stage of the current research. Specifically, the CLEI publication, written entirely in Spanish, was an initial exploration and proof-of-concept emphasizing the feasibility of employing remote sensing data for urban income estimation. It provided only preliminary results and a foundational methodological framework. Additionally, the CLEI paper was limited in scope, comprising only 10 pages including figures.

In contrast, the manuscript submitted to PLOS ONE constitutes a substantial and novel advancement of that initial work, extending to almost 30 pages without figures, reflecting the substantial changes and advancements between both manuscripts. Key differences and enhancements include:

- Methodological Refinements: The methodology has significantly evolved, incorporating a novel multi-resolution image stacking approach (combining 50x50m and 200x200m resolutions), which was entirely absent from the CLEI paper. This innovation significantly improved model accuracy, demonstrated by an R^2 of 0.878.

- Expanded Dataset: The current manuscript utilizes a substantially expanded dataset, incorporating images from multiple years (2013, 2018, and 2022). In contrast, the earlier publication focused solely on imagery from 2013, limiting its temporal robustness and scope.

- New Results and In-depth Analyses: The manuscript submitted to PLOS ONE introduces extensive new findings, including detailed temporal income estimations for 2018 and 2022, comprehensive qualitative case studies, and robust validations against census and survey data at multiple spatial aggregation levels. These critical analyses and results were not included in the CLEI publication.
- Enhanced Spatial Resolution and Validation: The current manuscript provides extensive validation and thorough discussion of the high-resolution (50x50m) estimations, presenting this core contribution for the first time in detail.
- Conceptual Contributions: Additionally, the current manuscript introduces substantial new conceptual insights regarding the measurement of income through remote sensing and broader considerations for model generalization and applicability.

Given these considerable methodological advancements, significantly expanded results, and new analytical depth, we believe the submitted manuscript represents a meaningful and distinct contribution beyond the initial CLEI conference publication. We have explicitly cited the earlier conference paper within the current manuscript to ensure complete transparency.

4. We note that you have indicated that there are restrictions to data sharing for this study. PLOS only allows data to be available upon request if there are legal or ethical restrictions on sharing data publicly.

Before we proceed with your manuscript, please address the following prompts:

a) If there are ethical or legal restrictions on sharing a de-identified data set, please explain them in detail (e.g., data contain potentially identifying or sensitive patient information, data are owned by a third-party organization, etc.) and who has imposed them (e.g., a Research Ethics Committee or Institutional Review Board, etc.). Please also provide contact information for a data access committee, ethics committee, or other institutional body to which data requests may be sent.

b) If there are no restrictions, please upload the minimal anonymized data set necessary to replicate your study findings to a stable, public repository and provide us with the relevant URLs, DOIs, or accession numbers. You also have the option of uploading the data as Supporting Information files, but we would recommend depositing data directly to a data repository if possible.

We will update your Data Availability statement on your behalf to reflect the information you provide.

Reply:

Thank you for highlighting this important issue regarding data availability.

The satellite imagery used in this study is proprietary and subject to legal restrictions imposed by third-party data providers, specifically Airbus DS Geo SA and the Argentine National Commission for Space Activities (CONAE). According to the end-user license agreement signed with these providers, we are not permitted to publicly share the raw satellite imagery.

Researchers interested in replicating the full analysis pipeline, starting from the original source imagery, must acquire this data commercially. The proprietary Pléiades and Pléiades NEO satellite imagery is owned by Airbus and can be purchased through their data portal: https://space-solutions.airbus.com/imagery/. To facilitate access, we provide unique product identifiers for each scene used in our study within the S1 Replication Package and S1 Replication Scripts. Researchers can use these identifiers to locate and purchase the exact imagery from Airbus.

However, we expect most researchers will primarily seek to replicate results either before model training (i.e., generating the small-area estimates) or the analytical outcomes derived from the model predictions (i.e., all analyses presented from section 6.1 onwards). To this end, we have made publicly available the necessary data and scripts for replicating these aspects of the study. These datasets are deposited in a Zenodo repository, and the corresponding replication scripts are available in a GitHub repository, both of which are cited in the Supporting Information. Specifically, we have provided:

- Argentina's 2010 Census and Survey Dataset: this dataset is used to replicate the small-area estimates employed in training our model alongside satellite images.

- Derived Income Estimates: Aggregated per capita income estimates at the census tract level (labels for CNN model training) and final 50x50m gridded income predictions for the Buenos Aires Metropolitan Area (years 2013, 2018, and 2022). These predictions enable the replication of all Figures and Tables presented in the Results section using scripts provided in the GitHub repository.

- Trained Model Weights: The EfficientNetV2 model weights (for both 4-band and 8-band inputs). Although these weights are not essential for replicating the primary findings, they are provided for further model fine-tuning in other contexts.

5. We note that Figures 1, 2, 3, 4, 5, 6, 7, 8, 9 11, 13 and 14 in your submission contain map/satellite images which may be copyrighted. All PLOS content is published under the Creative Commons Attribution License (CC BY 4.0), which means that the manuscript, images, and Supporting Information files will be freely available online, and any third party is permitted to access, download, copy, distribute, and use these materials in any way, even commercially, with proper attribution. For these reasons, we cannot publish previously copyrighted maps or satellite images created using proprietary data, such as Google software (Google Maps, Street View, and Earth).

We require you to either (1) present written permission from the copyright holder to publish these figures specifically under the CC BY 4.0 license, or (2) remove the figures from your submission.

Reply:

Thank you for your comment and for bringing this important matter to our attention. To address this concern, we have reviewed all the figures and updated some of them to be licencable under CC BY 4.0.

In our paper, we have created derived figures from IGN (Instituto Geográfico Nacional de La República Argeinta) aerial images in Figures 3, 4, 6, 9, and 12. The licence of these images is attached to the submission. According to the terms and conditions outlined on their website, "Commercial use is only permitted in the case of derivative works where the information is used as an input to generate a new product." We informally consulted with IGN, and they confirmed that the updated figures in this paper qualify as "new products," making them eligible for licensing under CC-BY 4.0. We have also ensured that the appropriate attribution is mentioned in the legend of each figure. Figures 2 and 4 from the original manuscript were removed to avoid any conflicting licensing issues.

Additionally, all maps included in this paper are either originally created by the authors (Figures 1, 7, 11, and S1) or generated using delimitations provided by INDEC (Instituto Nacional de Estadísticas y Censos) (Figures 2 and 7). INDEC adheres to Argentinian Open Data law (see their disclaimer here: https://www.indec.gob.ar/indec/web/Instit Indec-Transparencia-1). This law (Ley N° 27275) grants users, under Article 2, the right to access, reprocess, reuse, and redistribute this information. It states, "The right of access to public information includes the ability to freely search for, access, [...], reprocess, reuse, and redistribute information held by the obligated entities listed in Article 7 of this law [...]".

Finally, Figure 5 uses a background image from UGSG/LANDSAT, which is redistributable under appropriate attribution.

Reviewer # 1

The article proposes a novel and effective application area for satellite imagery, offering significant and impactful outcomes in this context.

Reply:

We would first like to thank the referee for their time and effort into crafting a very useful report. We greatly appreciate it as well as their overall positive view on our paper. In what follows we will attempt to describe how we sought to address each of their concerns into our revised manuscript.

1. The EfficientNetV2 architecture was selected due to its ability to deliver high performance despite a relatively low number of parameters, making it an efficient choice. However, the lack of a detailed comparison with alternative architectures can be considered a point open to critique.

Reply:

We appreciate this comment and agree that a detailed comparison with alternative architectures could be a point of further investigation. We discussed this possibility extensively. However, we decided against conducting extensive comparative testing with additional model architectures for the following reasons:

- **Focus of the Paper**: Our primary aim is, besides creating high-resolution estimates for Buenos Aires, to present a replicable methodology for satellite-based income estimation using widely available data sources (census/survey data and satellite imagery), connecting the small area estimation literature from economics with the current computer vision models applications. An exhaustive comparison of specific architectures would shift focus from this core methodological contribution to a detailed applied-AI paper.
- **Methodological Independence**: The proposed methodology is independent of the specific vision model architecture chosen. The core contribution—linking small area estimation outputs with satellite imagery to train a predictive model—can be implemented with various suitable deep learning architectures.
- **Rapidly Evolving Field**: The field of computer vision models is advancing at a remarkable pace. Any "optimal" architecture chosen today might be superseded quickly. For instance, compared to the time when the first drafts of this paper were written, lightweight transformer-based models are outperforming CNNs for many vision tasks. Continuously chasing the state-of-the-art architec-

ture would make the specific choice quickly obsolete and significantly expand the hyperparameter tuning space (considering that we also tested for image years, sizes, stacks, bands, learning rates), which is computationally intensive. Each model run already took approximately 52 hours on our available hardware (GeForce RTX 2060 Super GPU), as mentioned in Section 5.

We believe that focusing on the EfficientNetV2, a well-established and efficient architecture, allows us to clearly demonstrate the viability and effectiveness of our proposed pipeline without getting overly sidetracked by exhaustive architectural comparisons, which would be a substantial research effort in itself.

2. Although this high resolution allows for the creation of more detailed income maps, it should be noted that the generalization capacity of the study may be limited due to its reliance on non-public data available only for specific years.

Reply:

Thank you for highlighting this important point. We acknowledge that the Pléiades Neo imagery used is proprietary. We have added a footnote in the introduction (Footnote 1, page 2) and reiterated it in Section 4.2 (pages 8-9) to clarify this: "Although the imagery used in this study is proprietary, many governments already have access to high-resolution satellite images through partnerships with imagery providers, and frequently utilize them for diverse applications beyond AI-based predictions, such as urban planning, infrastructure monitoring, environmental assessments, and disaster management."

While individual researchers might face access limitations, the relevance for governmental and institutional bodies who often possess such data remains high. Our methodology provides a pathway for them to leverage these existing resources for socioeconomic estimation.

The training, validation, and test sets were separated by considering the different characteristics of urban areas. However, low-density population regions were excluded from the analysis, which may make it challenging to generalize the results to the entire population base.

Reply:

We agree that addressing low-density population regions presents a unique challenge. In the revised manuscript, we have explicitly acknowledged this limitation in Section 8 ("Generalizing the model to other cities"), with the following text:

"Secondly, it is crucial to emphasize that the current model does not discern whether

households actually exist within a given grid cell. Consequently, the model's predictions have limited utility in areas with sparse or no population, as such irrelevant data introduces noise into gradients and predictions. To address this shortcoming, implementing a two-stage modeling approach is advisable. The first stage would predict the presence of at least one household within the grid cell, while the second stage would subsequently estimate income levels only if the presence of housing is confirmed."

This highlights an area for future work to extend the model's applicability.

4. In this context, although the article presents a highly innovative method, further work is needed on generalization, model explainability, and ethical dimensions.

Reply:

Thank you for this valuable feedback. Given your comments as well as the other reviewers vision, we have substantially revised the manuscript to address these aspects. What was Section 7 in our previous manuscript (titled "Discussion") now is split into two different sections. Section 7 now focuses on the conceptual framework underpinning the measurement task (i.e., what is being measured), while Section 8 addresses the application of this model to other domains, such as different cities or regions. The generalization aspect is explored in detail in Section 8, specifically addressing methods like fine-tuning for individual cities and improving the model's performance using domain-specific information. Although model explainability and ethical considerations are not covered extensively—since doing so would require substantial restructuring—they are briefly discussed at the end of Section 8, in the following paragraph:

"The primary potential application for datasets generated by models such as this lies in the measurement of localized development, offering valuable insights for urban planning and the design of targeted interventions aimed at improving infrastructure or services. However, it is crucial to emphasize that these models, at least in their current state of development and validation, should not be directly used for high-stakes individual-level decisions such as taxation or determining eligibility for social programs without extremely careful consideration of the ethical aspects involved. Beyond these specific applications, broader ethical implications must be thoroughly addressed. Biases present in the original census or survey data, or in the small area estimation process itself, could be perpetuated or even amplified by the model, potentially leading to unfair resource allocation or misrepresentation of vulnerable communities. Privacy concerns, though somewhat mitigated by the use of aggregated census tract data for training, remain pertinent. There is also the risk of misuse, where such granular income data could be leveraged for discriminatory prac-

tices, such as redlining or targeted marketing that exploits socio-economic vulnerabilities. Therefore, the deployment of this technology must be accompanied by robust frameworks for ethical oversight, including transparency regarding model limitations, the incorporation of methods for model explainability to understand how predictions are made, ongoing bias audits, community engagement in areas where the data is applied, and a clear commitment to using these powerful tools to foster equitable development and well-being."

5. There is no reference from the year 2024 among the sources cited in the article. The references cover works published between 2010 and 2023. This could be considered a point to evaluate in terms of potential gaps or limitations in the coverage of the most current literature.

Reply:

Thank you for raising this point. Indeed, we had not previously included references from 2024 due to a lack of directly related papers published that year. Most of advancements in AI-based models for predicting socio-economic indicators via satellite imagery were focused on the use of transformers, mostly for images of much lower resolution than our method. We have nevertheless included updated references in the Related Literature section to make the reader aware of this recent trend.

6. In terms of potential enhancements, I suggest considering alternative measures for assessing income inequality and poverty indicators. For example, Theil Index and Atkinson Index could provide additional perspectives on income inequality, capturing variations and sensitivities that may complement the Gini coefficient analysis. Furthermore, alternative poverty measures, such as Absolute and Relative Poverty Rates, Headcount Index, and Poverty Gap Index, may offer a more comprehensive view of poverty depth and distribution characteristics. Incorporating these could broaden the analytical scope and contribute to a richer interpretation of the data.

While these measures are explained in your analysis, I would recommend providing further elaboration to enhance their clarity and impact. A more detailed breakdown of how each indicator—such as the 90/10 and 50/10 Ratios for capturing disparities across income segments, the Gini Coefficient for summarizing overall inequality, and the FGT indices (FGT(0), FGT(1), and FGT(2)) for measuring poverty dimensions—uniquely contributes to understanding inequality and poverty would enrich readers' grasp of the study's analytical depth and context.

Reply:

We appreciate this suggestion to broaden our analysis of inequality and poverty. We have now included the Theil Index and Atkinson Indices (with epsilon parameters of 1 and 2) in our analysis. These have been added to Table 4. We chose Atkinson (1) and (2) to illustrate how different degrees of inequality aversion (i.e., placing more weight on inequality at the lower end of the income distribution) affect the measure. The overall conclusions regarding the underestimation of inequality by satellite-based estimates compared to survey data, yet showing higher inequality than small area estimates alone, remain consistent. For brevity, we did not include these indicators in Figures 12 and 15, as the trends depicted the same patterns as those for the other indicators.

We would like to highlight that the Headcount Index and Poverty Gap Index were already included in our original manuscript, represented by the Foster-Greer-Thorbecke (FGT) indicators FGT(0) and FGT(1), respectively. We agree that not mentioning this explicitly was lacking in the original manuscript, as most readers are familiar with standard poverty measures but not so with the FGT indicators. We have not included relative poverty measures as their insights are well-captured by broader inequality indicators.

To enhance clarity and impact, as suggested, we have added a more detailed explanatory paragraph in Section 6.3 (pages 18-19) to help readers, especially those not specializing in economics, understand the nuances of each indicator used:

"We compare the following indicators of income distribution (for a detailed discussion on each indicator, refer to [1]): mean and median incomes; the 90/10 and 50/10 Ratios; the Gini coefficient; the Theil index; the Atkinson-1 and -2 indices; and the Foster-Greer-Thorbecke (FGT) poverty indicators (FGT(0), FGT(1), and FGT(2)). Mean and median incomes provide a sense of the typical income level. The mean gives the average, while the median represents the income of the middle household, which is less affected by the high skewness of the income distribution—as there are many households with low income but a few with very-high income—, and thus better reflects the conditions of the 'central' household. The 90/10 and 50/10 Ratios specifically highlight the spread of the income distribution by comparing different segments. The 90/10 ratio shows the disparity between the income of the household at the top and the bottom 10% of the income, while the 50/10 ratio illuminates the gap between the median household's income and the bottom 10% income. These ratios are useful for understanding how different parts of the income spectrum relate to each other, offering more granularity than a single summary statistic. Nevertheless, to obtain a more nuanced vision of the income distribution, economists often use indicators that depend not only on the income of a particular household but on every single one. The Gini coefficient provides a comprehensive single num-

ber that summarizes overall relative income inequality across the entire population. While widely used for its conciseness in comparing inequality levels, it's important to note that different income distributions can yield the same Gini coefficient, which is why it's complemented by other measures that reveal more about the shape of the distribution. The Theil index is another measure of relative inequality. Its unique strength lies in its decomposability, meaning it can be broken down to show how much inequality is due to differences between pre-defined groups (e.g., urban vs. rural, different education levels) versus inequality within those groups; while we don't perform such decomposition in this specific table, its inclusion represents a standard tool for deeper inequality analysis [2]. On the same line, the Atkinson indices (e.g., Atkinson (1) and Atkinson (2) in our table) measure inequality but uniquely incorporate a concept of 'inequality aversion.' This means they explicitly reflect how much a society might care about incomes at the lower end of the distribution, with different parameters of the Atkinson index (represented by the numbers in parentheses) allowing for varying degrees of emphasis on the poorest, making it a flexible tool for welfare-based inequality assessment [3]. Finally, we focus on absolute poverty indicators. The Foster-Greer-Thorbecke (FGT) family of poverty indicators offers a versatile framework for measuring different dimensions of poverty, calculated with the national poverty line from [4]: FGT(0), the poverty headcount ratio, is the most straightforward measure, indicating the percentage of the population whose income falls below a defined poverty line; FGT(1), the poverty gap index, goes beyond mere incidence to measure the depth of poverty, reflecting the average amount by which poor households' incomes fall short of the poverty line; and FGT(2), the squared poverty gap index, measures the severity of poverty, giving greater weight to those who are furthest below the poverty line and thus being particularly sensitive to the plight of the poorest among the poor [5]."

Reviewer # 2

While the study effectively demonstrates the use of deep learning and satellite imagery for income estimation and provides valuable insights, I have some concerns that, if addressed, could strengthen the work further.

Reply:

We sincerely thank Reviewer 2 for their thoughtful and constructive feedback. Their insightful comments have been invaluable in strengthening the manuscript, and we greatly appreciate the opportunity to improve our work.

1. The training relies on the census and survey data from 2010, which may not fully capture current or latest economic conditions.

Reply:

Thank you for raising this point. The choice of 2010 data was dictated by the availability of the most recent census microdata at the household level in Argentina necessary for the small area estimation (SAE) technique. In fact, while a new census was conducted in 2022, the household-level microdata required for our SAE approach has not yet been released. A key motivation for our methodology is precisely to bridge such data gaps. By training the model on historical relationships between visual features and income (from 2010), we can then apply it to more recent satellite imagery (e.g., 2013, 2018, and 2022 in our study) to estimate income distributions for periods where granular ground truth income data is unavailable. This allows us to infer more current economic conditions based on observable changes in the urban landscape, as discussed in Section 7. Nevertheless, it remains to be determined in future investigations the extent to which these estimates over time are accurate compared to the small area estimates ten years apart from the initial training data.

2. Since the model performance depends on similarities between training and prediction areas; fine-tuning is required for new regions.

Reply:

We fully agree with you. The generalization of the model to new regions is a critical aspect and was underdeveloped in the original manuscript. We have significantly expanded our discussion on this in the revised manuscript by splitting the previous section 7 (titled "Discussion") into two sections that consider two different aspects:

Section 7 ("Conceptualizing the Model's Measurement of Income") focuses upon

what the model measures, which is relevant for understanding its transferability. This has to do with the next comments.

Section 8 ("Generalizing the model to other cities") now elaborates on the requirements to apply this model to other domains via fine-tuning. Here we discuss how a model pre-trained on Buenos Aires data can be adapted by retraining its later layers with image-income pairs from the new target city. This is crucial because visual cues for income can vary significantly across different urban, cultural, and economic contexts. We emphasize that fine-tuning is essential not only for adapting to different visual features but also for calibrating income levels, as similar-looking urban environments can correspond to vastly different absolute income levels across cities with different economic profiles. For example, similar buildings in Buenos Aires compared to cities with different mean income like Rio de Janeiro, Tokio, Mumbai or New York City. Finally, potential improvements in the application to other domains, as well as key limitations, are discussed in this section. Some of its content comes from the original manuscript, but most of it is new.

3. The claim that the model can predict income for different years without updated data feels like a stretch. While it might catch long-term trends, incorporating short-term factors like inflation or employment changes would likely improve accuracy over time. More justification including explanations are required on this specific aspect.

Reply:

Thank you for this critical point. We have refined our claims and provided more justification in the revised manuscript, particularly in Section 7 ("Conceptualizing the Model's Measurement of Income"). We now explicitly argue that our model, by relying on structural features visible in satellite imagery (housing quality, infrastructure, urban layout), is more likely capturing "permanent income" rather than current, often volatile, income. Permanent income, as conceptualized by Friedman (1957) and more recently discussed by others (e.g., Meghir, 2004; Romer, 2019), reflects long-term average economic well-being and is less susceptible to short-term fluctuations like temporary unemployment or seasonal income changes.

Therefore, while the model can detect changes in urban fabric over time that correlate with shifts in long-term economic status, it is not designed to capture high-frequency economic shocks (like sudden inflation spikes or economic downturns) that don't immediately manifest in the physical environment. We acknowledge this limitation and clarify that our approach is geared towards understanding longer-term trends in spatial income distribution and development.

4. Other Uncertainties: The model seems to lean heavily on visual features for predicting income, but this could overlook key socio-economic factors like migration. This might lead to biased results, especially in similar-looking/or developing neighborhoods.

Reply:

Thank you for your insightful comment—we agree with this observation. The model indeed primarily leverages visual features derived from satellite imagery and, therefore, inherently overlooks certain crucial socio-economic factors such as migration patterns, which cannot easily be captured through imagery alone. We address this issue more explicitly in the updated Section 8, where we explore methods to generalize the model and clearly discuss its limitations. Specifically, we propose incorporating additional socio-economic variables into the final fully connected layers of the model, as demonstrated by Khachiyan (2022), alongside fine-tuning strategies tailored to specific urban contexts.

However, it is important to acknowledge that some socio-economic nuances will inevitably be missed by purely imagery-based approaches. Yet these limitations are not unique to our model but are common to high-resolution spatial income estimation methods in general, including traditional small-area estimation techniques and nighttime lights data. Despite these inherent constraints, our approach leverages the intuitive and empirically supported assumption that, on the long run, households with higher incomes (i.e. a higher permanent income) tend to reside in neighborhoods characterized by better infrastructure, more extensive public services, and superior overall living conditions. Even more so, lower-income households typically live in more homogeneous neighborhoods, as they have fewer choices in residential location due to economic constraints. Our model leverages this observable correlation between neighborhood characteristics and income levels, which is evident when comparing satellite imagery with census-based income data.

5. In addition, the model's sensitivity to times of day, vegetation, shadows, or the satellite's angle suggests that external conditions can also affect model predictions. Thus, the errors associated with the image data and subsequent image processing too can affect the results. In particular, if using any temporal data, the errors can add up.

Reply:

We agree with this key comment. The model indeed demonstrates sensitivity to external conditions such as vegetation changes, shadows, time of day, and satellite angles, all of which can introduce variability into the predictions. On one hand, the sensitivity to varying conditions such as the hour of the day, the vegetation, shadows or the satellite angle could be easily solved by employing a larger dataset—something that was not possible due to our limited access to data at only these three points in time. On the other hand, we could have tried to further improve the model performance by including ad-hoc information. As mentioned in the previous comment and in Section 8, Khachiyan (2022) does this to greatly improve the model precision and reduce the variance over predictions. Nevertheless, our goal was to establish a robust methodological framework for high-resolution income estimation using solely satellite imagery, rather than achieving the absolute best estimates specifically for Buenos Aires. We did this because, by having such a strong base model for predicting income, the user can focus on fine-tuning and including the variables relevant to the specific domain, instead on proposing a highly specific solution for Buenos Aires that might not work for other cities.

Furthermore, your observation about cumulative errors over time is particularly relevant. Temporal errors or changing contextual factors——such as shifts in architectural trends or new public housing policies—could mislead the model if the training data lacks similar precedents. Given these dynamics, we stress the importance of continuous model evaluation to ensure long-term stability and accuracy, as spatially disaggregated socioeconomic estimation models must evolve alongside the phenomena they aim to capture.

6. Temporal constraints: The methodology has limited ability to detect short-term economic fluctuations; the model primarily captures structural and spatial variations. It is not clear how short-term fluctuations can be captured.

Reply:

We agree with this fact. As elaborated in our response to Comment 3 and in Section 7, our model is designed to capture changes related to long-term or permanent income, which manifest in the physical urban environment. It is not well-suited for detecting short-term economic fluctuations that do not have immediate visual correlates in satellite imagery. We have explicitly clarified this focus in the revised manuscript to set appropriate expectations for the model's capabilities. Capturing short-term fluctuations would likely require integrating different types of data (e.g., real-time

economic indicators, high-frequency survey data) beyond the scope of the current satellite-imagery-based approach.

7. Images: The authors used an approach that combines multiple image sizes (e.g., 50x50m and 200x200m) which improves context but could introduce noise. More explanation may be provided as to how the noise was reduced.

Reply:

Thank you for highlighting the importance of explaining how our multi-scale image stacking approach helps reduce noise and improve predictions. We agree that our initial mention was too vague and that it required further development. To address this, we have added a pargraph in the main script (Section 4.2, page 10) that elaborates on this. This paragraph references new supplementary material, specifically Figure S1 and Table S1, which visually and quantitatively demonstrate the improvement.

Figure S1 provides a qualitative comparison: both its panels show the model's 50x50 meter predictions for 2013. The left panel uses a model trained only with 50x50m images (unstacked), while the right panel uses our preferred model trained with stacked 50x50m + 200x200m images. While the general spatial income distribution is similar, the unstacked model (left) exhibits considerably lower spatial correlation, resulting in a 'noisier' map. In contrast, the stacked model (right) produces a map with higher spatial correlation, more closely resembling the spatial patterns of the Small Area Estimation data used for training (see Figure 13 in the manuscript). This observation holds for other time points and for the 50x50m+100x100m stacked configuration, and for both 2013, 2018, and 2022 as well.

To quantify this, we employ Moran's I, a measure of spatial autocorrelation [6]. This statistic ranges from -1 (perfect dispersion) to +1 (perfect correlation/clustering), with 0 indicating random spatial distribution. We calculated Moran's I using a uniform distanced-based binary band for various contiguity distances (100m, 500m, 1000m, and 1500m) for the Autonomous City of Buenos Aires. Due to computational constraints (the weight matrix for the entire dataset at 500m contiguity exceeded 64GB RAM, and it exponentially increases the further the distance considered), we limited the Moran's I calculation to this major urban core.

Table S1 (Supplementary Material) presents these Moran's I values. The original training data (Census Tract Average Income) exhibits high spatial autocorrelation, which gradually decreases with distance. The predictions from our preferred 50x50m+200x200m stacked model closely match this pattern of spatial autocorrelation across all distance thresholds. In contrast, models using unstacked images or a smaller context window (50x50m+100x100m) show significantly lower spatial autocorrelation, diverging more from the training data's characteristics. This indi-

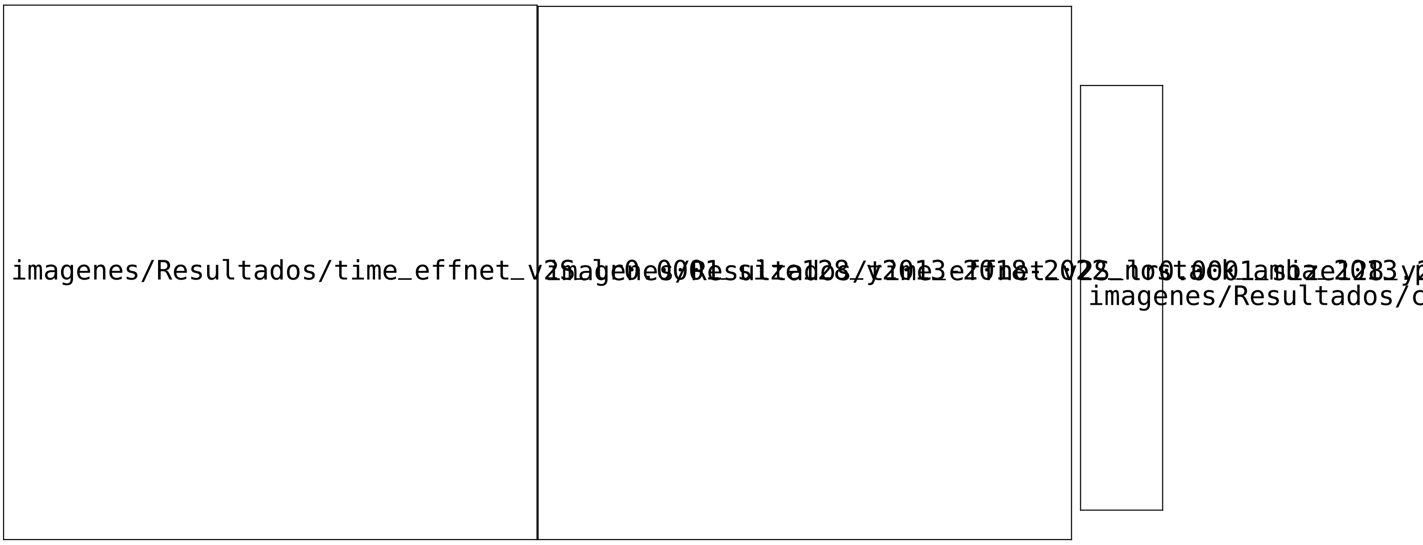

(a) 50 × 50 meters.

(b) 50 × 50 + 200 × 200 meters.

Figure S1. Spatial per capita income estimates of Buenos Aires in 2013 using unstacked and stacked images.

cates that the stacked approach, particularly with a larger context window, helps the model learn and reproduce more spatially coherent income patterns, effectively reducing noise and aligning better with the underlying socioeconomic landscape. This improved spatial consistency, alongside its superior MSE performance (Table 3), justifies our selection of the stacked model.

8. Broad results: Results are sensitive to urban patterns in Buenos Aires and might not generalize well to different cities. In essence, the model developed can be highly local. Since the model is trained on data from Buenos Aires, there's a risk of overfitting. Authors mention that the model can be applied to other cities, but might be overlooking some critical differences between cities—like cultural, economic, or geographical factors. Including more localized training data for each region could boost the accuracy. I suggest the authors elaborate on the discussion part on how the model can be fine-tuned to apply to other regions.

Table S1. Moran's I in the Autonomous City of Buenos Aires (2013) across Model Configurations and Distance Band Thresholds.

Variable		Distance Band (in meters) **100**	**500**	**1000**	**1500**
Census Tract Average Income		0.948	0.812	0.683	0.587
Model's Predictions	Stack 50x50 + 200x200	0.931	0.748	0.628	0.532
	Stack 50x50 + 100x100	0.846	0.628	0.512	0.428
	Unstacked 50x50	0.755	0.510	0.507	0.433

Reply:

This is a very important point, and we have expanded on it significantly in Section **??** ("Generalizing the model to other cities"). We explicitly state that the model trained on Buenos Aires is indeed localized and that direct application to other cities without adaptation would likely yield suboptimal results due to differing urban forms, architectural styles, and socio-economic contexts. We outline the necessity of fine-tuning using local data from the target region as the primary strategy for adaptation. This involves retraining parts of the network (especially the later, more specialized layers) with image-income pairs from the new city. Furthermore, for an application to other cities is key to ensure a proper equalization of income values, as similar buildings in different cities usually imply different household incomes. We also discuss the potential for incorporating local non-visual data to further enhance accuracy in new settings. As mentioned previously, our aim was to provide a foundational methodology that is adaptable, rather than a universally applicable off-the-shelf model.

8. Abstract may be clarified to include specific time periods or intervals of the study.

Reply:

Thank you. We have revised the abstract to explicitly mention that the income estimations are made for the years 2013, 2018, and 2022. The abstract now reads (in part): "...to estimate per capita income at a 50x50 meter resolution for 2013, 2018 and 2022."

References

[1] Ray D. Development Economics. Princeton University Press; 1998. Available from: http://www.jstor.org/stable/j.ctvcm4hmx.

[2] Theil H. The measurement of inequality by components of income. Economics Letters. 1979;2(2):197–199. doi:https://doi.org/10.1016/0165-1765(79)90173-3.

[3] Atkinson AB. On the measurement of inequality. Journal of Economic Theory. 1970;2(3):244–263. doi:https://doi.org/10.1016/0022-0531(70)90039-6.

[4] y Banco Mundial C. Socio-Economic Database for Latin America and the Caribbean. 2023;.

[5] Foster J, Greer J, Thorbecke E. A Class of Decomposable Poverty Measures. Econometrica. 1984;52(3):761. doi:10.2307/1913475.

[6] LeSage J, Pace RK. Introduction to Spatial Econometrics. Chapman and Hall/CRC. 2009;doi:https://doi.org/10.1201/9781420064254.
