# Supplementary material for: Deep learning with satellite images enables high-resolution income estimation: A case study of Buenos Aires
Source: PLoS One. 2026 Jan 16;21(1):e0338110. doi: 10.1371/journal.pone.0338110 (PMC12810835; doi:10.1371/journal.pone.0338110)
Supplement: Attachment — Submitted filename: Response to Reviewers - R2.pdf [file pone.0338110.s005.pdf]

Response to Reviewers

Manuscript ID: PONE-D-24-42689
Title: Deep learning with satellite images enables high-resolution income estimation: a case study of Buenos Aires

We sincerely thank the editor and reviewers for their time and valuable feedback. We were pleased to see that the reviewer found our revisions to be a great job and was satisfied with the level of detail provided. We have addressed the final minor suggestions as detailed below.

Reviewer #2

Minor Suggestion 1: In Table 3 the title ”Hyperparameter Configurations” seems misleading... I suggest renaming it to ”Model Configurations and Mean Squared Error...” or something similar.

We thank the reviewer for this suggestion. The original title was indeed not precise. We have revised the title of Table 3 to “Model Selection: Configurations and Mean Squared Error on the Test Set.” to more accurately reflect its contents.

Minor Suggestion (Figures) 1: For “Figure 1”: In “6. Results” for the ‘example income deciles map...’, including a scale bar, north arrow and coordinates would be ideal... If feasible, including an earlier figure that outlines the study area... would improve readability...

We agree completely that an earlier study area map would improve spatial context for the reader. We have created a new map of the study area, which is now included as the new Figure 1. This map contains a scale bar, north arrow, and coordinates as requested. The original flowchart has been renumbered to Figure 2.

Minor Suggestion (Figures) 2: For “Figure 2” – including a north arrow and the suffix for latitude and longitude would make the map more visually appealing.

Thank you for this suggestion. The figure (now renumbered) and all other maps have been updated to include north arrows and standard coordinate suffixes (e.g., °S, °W).

Minor Suggestion (Figures) 3: I suggest using a consistent symbol for meters throughout the manuscript. As is stands, ‘m’ is used in the manuscript. However ‘mts’ is used in Figure 3 and parts of manuscript interchangeably.

This is an important point for consistency. We have reviewed the entire manuscript and all figures and have standardized the unit for meters to ’m’ throughout.

Minor Suggestion (Figures) 4 & 5: "Figure 5" should include a scalebar, north arrow, and coordinates suffixes... "Figure 7" "a" seems cut off at the bottom... Inclusion of scalebar, coordinates, and suffixes would be help...

We appreciate the reviewer pointing these out. Figures 2, 3, 6, 8, and 12 have been updated to be self-explanatory with the inclusion of scale bars, north arrows, and coordinate suffixes. We have also corrected the formatting issue with the label in Figure 7 (now Figure 8). The blank region was not a missing label but an area with no available satellite imagery for that year, hence no predictions were made. We have now clarified that on the Figure, as well as in Figure 12 were the same thing happened.

Minor Suggestion (Figures) 6: "Figure 10" I suggest including R2 value in figure caption or image subplots.

This is a helpful suggestion to improve clarity. We have included those values in each of the subplots.
